# La FAM fatale: USP9X in development and disease

**DOI:** 10.1007/s00018-015-1851-0

**Published:** 2015-02-12

**Authors:** Mariyam Murtaza, Lachlan A. Jolly, Jozef Gecz, Stephen A. Wood

**Affiliations:** 1 The Eskitis Institute for Drug Discovery, Griffith University, Brisbane, QLD Australia; 2 School of Paediatrics and Reproductive Health and The Robinson Research Institute, The University of Adelaide, Adelaide, Australia

**Keywords:** Ubiquitin, Fat facets, Embryo, Stem cells

## Abstract

Deubiquitylating enzymes (DUBs), act downstream of ubiquitylation. As such, these post-post-translational modifiers function as the final arbitrators of a protein substrate’s ubiquitylation status, thus regulating its fate. In most instances, DUBs moderate the absolute level of a substrate, its locality or activity, rather than being an “all-or-none” phenomenon. Yet, disruption of this quantitative regulation can produce dramatic qualitative differences. The ubiquitin-specific protease 9X (USP9X/FAM) is a substrate-specific DUB, which displays an extraordinarily high level of sequence conservation from *Drosophila* to mammals. It is primarily the recent revelations of USP9X’s pivotal role in human cancers, both as oncogene or tumour suppressor, in developmental disorders including intellectual disability, epilepsy, autism and developmental delay that has led to a subsequent re-examination of its molecular and cellular functions. Results from experimental animal models have implicated USP9X in neurodegeneration, including Parkinson’s and Alzheimer’s disease, as well as autoimmune diseases. In this review, we describe the current and accumulated knowledge on the molecular, cellular and developmental aspects of USP9X function within the context of the biological consequences during normal development and disease.

## Introduction

A large part of tissue development regulation and homoeostasis concerns the precise control of cell numbers, morphology and function in response to local or systemic signals. These phenomena are regulated at multiple levels including post-translational modifications (PTMs), which determine protein function, including localisation, activity, stabilisation and fate. The key characteristics of PTMs are that they are rapid and reversible allowing cells to regulate signalling pathways in response to environmental conditions, through the quantitative regulation of substrate function. One of the best-studied PTMs is ubiquitylation in which proteins are covalently linked to the 76 amino acid protein, ubiquitin. Initially described as a tag for degradation, ubiquitylation has emerged as a dynamic and versatile PTM, which regulates most, if not all, cellular processes (see [1, 2] for reviews). Ubiquitylation is a reversible process where the deconjugation of ubiquitin is performed by a family of enzymes called deubiquitylases (DUBs).

There are approximately 95 DUBs encoded by the human genome [3]. The largest family is the ubiquitin-specific proteases (USP), which is substrate specific and precisely regulates cellular processes. This diverse family of proteins shares a catalytic domain of between 300 and 800 amino acids, which contain two short conserved cysteine and histidine catalytic motifs separated by long stretches of non-conserved intervening sequences. The catalytic domain is also flanked by non-conserved N- and/or C-terminal extensions, which impart substrate specificity [3, 4]. Apart from the conserved catalytic motifs, the USPs share little or no similarity; however, USP members perform in effect post-PTMs as their substrates are already post-translationally modified by ubiquitylation. Indeed, ubiquitylation itself is also often preceded by other PTMs such as phosphorylation [5] or hydroxylation [6]. Therefore, the activity and functional relevance of all USPs is highly context specific, dependent on the activity of the upstream enzymes, such as E3 ubiquitin ligase or kinases, performing the preceding PTMs. Despite residing at the end of a chain of PTMs, USPs are not redundant proteins as loss of function in nearly all USPs is associated with, often severe, functional consequences. In this review, we will use the example of USP9X to illustrate the relevance of studying this emerging family of ubiquitin-modifying proteins.

### USP9X is a highly conserved DUB

**Fig. 1:**
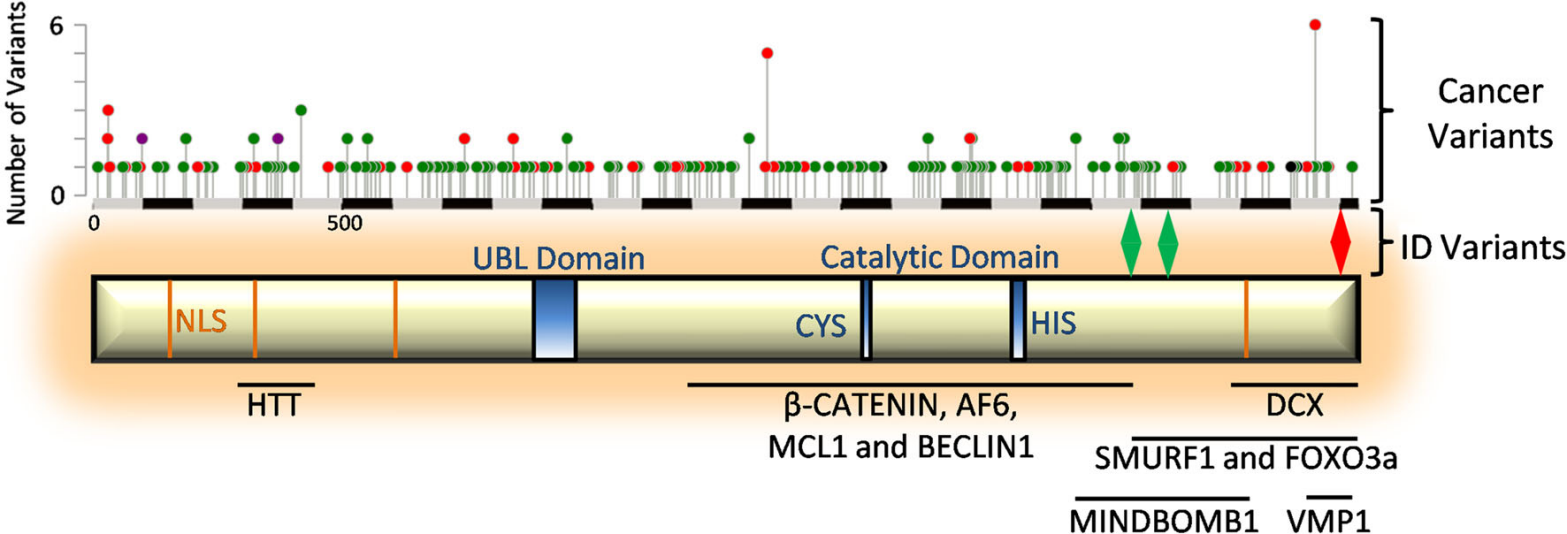
Structural information of USP9X. Schematic of USP9X structure showing functional domains and nuclear localisation sequence (NLS) motifs. Below the schematic are the regions of USP9X known to facilitate binding to the listed interacting proteins. Above the schematic is a scale (in amino acids), the localisation of variants associated with ID^18^ and a histogram of variants found in cancer samples (cBioportal). *Red* indicates nonsense variants, *green* represents missense variants and *purple* indicates both

Although USP9X is over 2,550 amino acids in size, very little is known about the protein structure. Apart from the USP-definitive cysteine and histidine box catalytic motifs, the only other recognisable domain is a ubiquitin-like module (Ubl) in the N-terminal extension (amino acids 886–970) (Fig. 1). It has been proposed that in solution, USP9X is an elongated monomeric protein [7]. Enzymatically, USP9X can cleave mono-ubiquitin from substrates and a wide variety of ubiquitin chains, including K48, K63 and K29 linkages, [8–13] and is known to interact with at least 35 proteins, many of which are substrates (Table 1). The protein sequence of USP9X does, however, display a remarkable degree of evolutionary conservation from *Drosophila* to mammals. Across vertebrates, the level of conservation of USP9X (>90 %) is equivalent to that of the developmental master-regulatory genes, such as Pax6 and β-catenin, and is maintained across the entire protein [14]. High sequence conservation of USP9X mirrors its function across species. The first *USP9X* homologue identified was the *Drosophila* gene *fat facets* (*faf*), found in a mutagenesis screen and shown to be required for the development of the syncytial stage embryo as well as photoreceptor fate determination [15]. The expression of mouse *Usp9x* rescued both eye and embryo defects in *faf* mutants, emphasising functional conservation from flies to mammals [16, 17]. In addition, the expression of human *USP9X* rescues axonal and migration defects in neurons derived from *Usp9x* conditionally deleted mice [18]. Thus, *USP9X* molecular functions are highly conserved throughout evolution. USP9X is in fact among the top 500 human genes with the lowest ‘tolerance’ to DNA variation [19] and exhibits selective constraint over evolution [20]. The discoveries of deleterious *USP9X* genetic variants found associated with neurological disorders [18] and enriched in cancer samples (Fig. 1 and below) are in line with the strong selection against USP9X mutation.

**Table 1:** List of substrates and proteins interacting with USP9X

Substrates	Major role of substrate	Interaction shown by	References
AF-6	Cell adhesion and polarity	Co-IP from brain lysates	[12, 109]
AGS3	Mitotic spindle orientation Golgi function	Tandem mass spectrometry, Co-IP from HEK293 and rat brain lysates	[27]
α-SYNUCLEIN	Synaptic maintenance Major component of Lewy bodies	Co-IP SH-SY5Y lysates and rat brain homogenates	[86]
ASK1	Apoptosis	Co-IP from HEK293A cells	[53]
BCL10	Activation of NFκB pathway	Co-IP JE6.1 cells	[110]
β-Catenin	Cell adhesion Wnt signalling	Co-IP from brain, L, MCF7 and T84 cells	[111]
EFA6	Cell adhesion and polarity	Co-IP from MDCK cell lysates	[23]
Epsin/liquid facets	Endocytosis Notch signalling	Co-IP from brain lysates, *Drosophila* eye disc protein extracts	[32, 35]
ErbB2	Oncogene	IP from SK-BR-3 cells	[10]
ERG	Transcription factor Prostate cancer	GST pulldowns and Co-IP from VCaP prostate cancer cells	[92]
ITCH	E3 Ligase	Co-IP from HEK293 and GST pulldown from brain lysates	[11, 37]
MARCH7	E3 Ligase Ubiquitin system	Sepharose pulldowns and IP from HEK293 cells	[112]
MARK4	Cell adhesion and polarity	Tandem affinity purification and Co-IP from HEK293 cells and brain lysates	[8]
MCL1	Apoptosis	Co-IP HEK293T cells	[24]
NUAK1	AMPK-related kinase	Tandem affinity purification and Co-IP from brain lysates and HEK293	[8]
PEX5	Peroxisomal protein shuttling	IP from rat liver and HeLa cell lysates	[7]
SMAD4	TGFβ signalling	Co-IP from HEK293	[9]
SMURF1	TGFβ/BMP signalling pathway	Quantitative mass spectrometry and Co-IP from HEK293 lysates	[65]
SMN	Maintenance of motor neurons	Mass spectrometry and Co-IP from HEK293 and HeLa cells	[42]
SURVIVIN	Mitosis Apoptosis	Co-IP from *Xenopus* oocytes and HeLa cells	[13]
VASA	Transcriptional regulation	Yeast two hybrid and tandem immunoprecipitation	[113]

Interactor	Major role of interactor	Interaction shown by	References
BAG1	Cell survival	Co-IP primary AML cells	[60]
DCX	Neuronal migration Trafficking	Co-IP from brain lysates	[82]
FOXO3	Transcription factor	IP from T74D breast cancer cell line	[114]
HDAC6	Transcriptional regulation	Mass spectrometry-based proteomics strategy	[115]
HUNTINGTIN	Trafficking Transcription	Yeast two-hybrid and Co-IP from brain	[88]
IKAP	Neurogenesis and cell migration	Mass spectrometry HEK293 cells	[116]
Imd	Innate immune response	Co-immunoprecipitation from S2 cells	[117]
Lebercilin	Leber congenital amaurosis	Quantitative protein complex analysis in HEK293 cells	[118]
Mind bomb1	E3 Ligase Signalling	GST pulldown from neuronal lysates, yeast two-hybrid screening and Co-IP	[39, 40]
p100	TNFa signalling	Tandem affinity purification	[119]
SALL4	Self-renewal	Mass spectrometry and protein interaction networks	[75]
SOX2	Transcription factor with critical roles in embryonic CNS development	Multi-dimensional protein identification technology in medulloblastoma cell line	[74]
TANK	Type I interferon induction	Mass spectrometry and protein interaction network	[120]
VMP1	Autophagy	Co-IP AR42J acinar cell lysates	[121]

## Cellular functions of USP9X

Knowing USP9X’s cellular functions is a prerequisite for the understanding of its role in development and disease. Here, we highlight the diverse and dynamic subcellular localisation of USP9X before illustrating its involvement in cellular processes that are fundamental to many aspects of development and disease, namely protein trafficking, cell polarity and cell death.

### Cellular localisation of USP9X

The intracellular localisation of USP9X is complex, dynamic and dependent on cell type and status. In general, however, immunofluorescence on both cultured and in vivo cells detects USP9X predominantly in cytoplasmic and membrane-associated puncta [21–23]. In polarised T84 epithelial cells, USP9X-labelled puncta represented sites of vesicular protein trafficking, such as the Golgi apparatus, late endosomes and other cytoplasmic vesicles [22]. In other epithelial cell lines (MDCK cells), USP9X has also been detected localised at the cell membrane at the primordial [23] and mature [12] adhesion junctions. Other studies report small portions of USP9X present at the mitochondria [24] and in the nucleus [25, 26] as well as a significant portion in the cytoplasm [22]. USP9X subcellular localisation is dynamic. For example, disrupting protein trafficking or the Golgi in polarised epithelia results in relocation or accumulation of USP9X, suggesting that it circulates between a number of organelles and vesicles with different resident times at each compartment. Conversely, expressing cadherin cell adhesion molecules in fibroblasts altered USP9X localisation [22]. The existence of multiple, dynamic pools is consistent with a variety of cellular USP9X functions regulated by localisation, but remains poorly characterised. In addition, several USP9X substrates, for example, EFA6 and AGS3, are also present in multiple cellular localisations and only a subset of USP9X and its substrate show spatial colocalisation [23, 27]. These observations highlight the need to interrogate USP9X/substrate interactions using techniques, which distinguish specific subcellular compartments as opposed to more disruptive biochemical approaches used to date where cellular architecture is destroyed.

### USP9X in protein trafficking/endocytosis

Directed protein trafficking through the vesicular network controls many aspects of cellular morphology, function and signalling. It is regulated at multiple levels by ubiquitylation, in particular mono-ubiquitylation, which occurs in both *cis* and *trans* [28–30]. In cis, the ubiquitylation status of cargo proteins (e.g. ligand-activated receptor tyrosine kinases) can direct vesicular trafficking pathways and protein fate, whilst in *trans* the mono- and poly-ubiquitylation of vesicular adaptor proteins (e.g. the endocytic adaptor Epsin) also regulates vesicular transport machinery. Not surprisingly, the role of DUBs in protein trafficking events has now been recognised [26, 30].

In *Drosophila*, genetic screening identified *liquid facets (lqf)* as *faf*’s critical substrate in photoreceptor fate determination [31]. *lqf* is the *Drosophila* homologue of *Epsin,* an endocytic adaptor protein involved in both clathrin-mediated and clathrin-independent endocytosis. Faf opposes the ubiquitin–proteasome mediated degradation of Lqf [32]. A disruption in the Delta/Notch signalling events underlies the eye phenotype observed in *faf* and *lqf* null mutants [33, 34]. Faf regulation of Lqf is essential for a specific endocytic event (as opposed to bulk endocytosis) required for the competence of the Notch ligand Delta to signal [34]. The Usp9x–Epsin interaction has also been maintained in mammals. Usp9x and Epsin co-immunoprecipitate from synapses in the rat brain, and evidence suggests that this interaction is promoted by neuronal activity resulting in specific deubiquitylation of Epsin [35].

USP9X also regulates protein trafficking by deubiquitylation of multiple endocytic E3 ligases. The E3 ligase Itch antagonises the internalisation and trafficking of activated epidermal growth factor receptor (EGFR) to the lysosome by promoting the proteasomal degradation of the endocytic accessory protein endophilin and another E3 ligase, Cbl [36–38]. However, Itch also auto-ubiquitylates the following EGFR activation and requires specific deubiquitylation by USP9X to protect itself [11]. Similarly, Mind bomb’s E3 ubiquitin ligase activity is required for Delta endocytosis and signalling through Notch, and Mind bomb also displays auto-ubiquitylating activity and degradation at the proteasome. USP9X and Mind bomb1 interaction has been shown previously [39, 40]. Interestingly, Mind bomb1 ubiquitylates and catalyses the proteasomal degradation of SMN [41], a USP9X substrate [42]. These findings indicate that it is possible for USP9X and Mind bomb1 to cooperate in the regulation of SMN protein and in Mind bomb1-regulated endocytic events.

Finally, USP9X is implicated in the regulation of endocytosis of the breast cancer oncogene and EGFR family member ErbB2. In SK-BR3 cells (breast cancer cells overexpressing ErbB2) treated with the proteasome inhibitor bortezomib, ErbB2 co-immunoprecipitates with a complex containing c-Cbl and USP9X. Reduction in USP9X levels increases bortezomib-induced downregulation of ErbB2, suggesting that USP9X is associated with the internalisation and ubiquitylation status of ErbB2 [10]. These data support the roles of USP9X in endocytosis and downstream events. However, USP9X also colocalises with markers of the trans-Golgi network in multiple cell types [22]. The localisation of proteins associated with the late Golgi compartment is affected in USP9X knockdown cells possibly by regulating the levels of AGS3 [27]. Furthermore, it is proposed that USP9X regulates the trafficking of the E-cadherin/β-catenin dimer in epithelial cells undergoing polarisation [22]. Collectively, these studies strongly suggest that USP9X plays an important role in the regulation of protein trafficking at multiple points in the cell.

### USP9X regulation of polarity

Cell polarity underpins many aspects of normal tissue growth and morphogenesis, and deregulation is a feature of many disorders, including tumourogenesis and metastasis. There are two types of polarity: apical–basal and planar cell polarity (PCP), and USP9X is implicated in both. PCP is the polarisation of epithelial cells within the plane orthogonal to their apical–basal axes. Core PCP proteins instruct these polarised cell movements [43], and *Drosophila* genetics has revealed that *faf* acts upstream in this process [44]. Apical–basal polarity in epithelia is established and maintained via three inter-dependent processes: cell–cell adhesion, activated cell polarity complexes and polarised protein trafficking. The establishment of adherens junctions (AJs) and subsequently tight Junctions (TJs) is considered the pioneering event and multiple studies have implicated USP9X in this process. Depleting USP9X disrupts TJ assembly in MDCK epithelial cells, an effect mediated through EFA6. In these cells, USP9X is required to deubiquitylate EFA6 resulting in localised stabilisation of EFA6, thereby promoting de novo assembly of TJs [23, 45]. USP9X also co-localises and binds to AF-6, a critical regulator of intercellular junctions and a similar colocalisation is observed in the developing eye [12, 46]. In pre-implantation embryos, USP9X depletion results in a failure of blastomere compaction, which is associated with mislocalisation of AF6 to apical surfaces, indicative of a polarity defect [47]. In the highly polarised neural progenitor cells of the developing brain, USP9X is apically enriched and again partially colocalises with cell junction proteins β-catenin and N-cadherin. Overexpression of USP9X in embryonic stem cell (ESC)-derived neural progenitors promoted their polarisation in vitro [21]. Other studies describe USP9X in the regulation of adhesion junctions more generally, with indirect implications for polarity. For example, USP9X also colocalises with adhesion proteins such as β-catenin, p120 catenin, E-cadherin and ZO-1 in the polarised human intestinal cell line T84. Interestingly, USP9X physically interacts with β-catenin (a known substrate) and E-cadherin only in subconfluent cells where adhesion junctions are undergoing dynamic rearrangements, and not in confluent cells exhibiting mature adhesion junctions [22]. In MDCK cells, overexpression of the USP9X catalytic domain increased the steady-state levels of β-catenin, presumably by its ability to deubiquitylate and hence rescue from proteasomal degradation. However, a partial knockdown of USP9X did not affect the protein levels of β-catenin [23], suggesting a concentration- dependent role for USP9X–β-catenin regulation. Together, these studies show that USP9X interacts with multiple cell adhesion proteins, and that cell adhesion (and polarity) is itself sensitive to changes in USP9X expression.

Downstream of adhesion, cell polarity complexes are activated that orchestrate the establishment and maintenance of apical–basal polarity. Genetic and molecular evidence has revealed the importance of the highly conserved Par family of proteins as components of these polarity complexes [48]. USP9X binds to the AMP-activated protein kinase (AMPK)-related kinases NUAK1 and MARK4 (Par-1 homologues) [8, 49]. The family of AMPK-related kinases are activated by the LKB1 kinase (Par-4 homologue), and are known to regulate polarity [50]. NUAK1 and MARK4 are substrates of USP9X, and the resultant deubiquitylation of these two kinases promotes their phosphorylation and activation by LKB1 [8], suggesting USP9X might regulate their activities during the establishment and maintenance of polarity. Further evidence suggests that USP9X is also important for polarised vesicular trafficking (see above). Together, these studies reveal that USP9X is important for polarity, interacting genetically with core PCP genes and molecularly at multiple stages of apical–basal polarity, including establishment of cell adhesion complexes and the regulation of polarity complexes.

### USP9X and cell death

Apoptosis is an integral part of development and disease and involves a highly regulated sequence of events. USP9X has been shown to regulate both pro- and anti-apoptotic pathways. Regarding its pro-apoptotic activities, three independent lines of evidence implicate USP9X. In *Drosophila*, *faf* has been identified as a dominant enhancer of apoptosis induced by either the Grim or Reaper proteins [51, 52]. Coexpression of *Reaper* or *Grim* with *faf* in the *Drosophila* CNS midline results in a dramatic reduction in midline glia and neurons [52] indicative of a pro-apoptotic role for *faf*. In mammalian cells, USP9X has been shown to regulate the levels of stress-sensing pro-apoptotic kinases that initiate the apoptotic JNK signalling cascade. In response to extracellular and intracellular stress (for example, oxidative stress), the pro-apoptopic kinase, apoptosis signal-regulating kinase1 (ASK-1), is activated resulting in the selective activation of the JNK and p38 MAPK pathways. USP9X binds ASK-1 and protects it from proteasomal degradation. In USP9X-depleted cells, activation of JNK and p38 was reduced and oxidative stress-induced cell death was decreased [53]. A similar role for USP9X was also found in the regulation of DLK kinase, which is activated and stabilised (by USP9X) at sites of stress in neurons (e.g. injury or removal of trophic support), enabling it to activate pro-apoptotic JNK signalling [54].

Conversely, USP9X is known to promote the activities of anti-apoptotic factors, MCL1 and Survivin. MCL1 is a member of the anti-apoptotic BCL2 family that is essential for promoting the survival of multiple cell types [55–57]. USP9X deubiquitylates poly-ubiquitylated MCL1, protecting it from proteasomal degradation, thus increasing its stability and thereby promoting cell survival. USP9X depletion increases poly-ubiquitylation of MCL1, enhances its turnover and sensitises tumour cell lines to the pro-apoptotic drug ABT-737 (a BCL2 antagonist that does not target MCL1) [24]. Interestingly, synergistic enhancement of the pro-apoptotic effects of ABT-737 also occurs when used in combination with gemcitabine, the addition of which was specifically associated with a disruption of the USP9X–MCL1 interaction [58]. Increased MCL1 turnover and sensitivity to apoptotic stimuli is also reported in chronic myelogenous leukaemia (CML) cells following USP9X depletion [59]. In addition, studies conducted in acute myeloid leukaemia (AML) cells, the anti-apoptotic activity of the BCL2-associated athanogene-1 (BAG1) was suggested to occur via USP9X and MCL1; BAG1 was found to immunoprecipitate with both USP9X and MCL1, and reduced BAG1 expression resulted in the depletion of both and associated with elevated MCL1 ubiquitylation [60]. Thus in multiple cancer cell types, the level at which USP9X is able to deubiquitylate and stabilise MCL1 dictates its anti-apoptotic function. USP9X also interacts with the anti-apoptotic factor survivin [13, 61], although the relevance of this interaction in the context of apoptosis has not been addressed. Collectively, the data suggest that USP9X can function at multiple stages during initiation and execution of the pathways involved in cell death. It can display both pro- and anti-cell death functions, mediated by the ability of USP9X to deubiquitylate the critical components of the apoptotic signalling networks.

## USP9X in development

The initial investigations of Faf showed that it was required for cell fate decisions of the developing eye [15]. The discovery of *Usp9x* in mouse was the result of gene-trap screen of genes expressed during early embryonic development [17]. Thus from its origins, USP9X has been implicated in developmental processes and recent investigations have identified multiple requirements during embryogenesis, with particular focus on USP9X’s roles in stem cells and neural development.

### USP9X in embryogenesis

*Usp9x* is essential for embryogenesis as both *Usp9x* ^−*/y*^ males and even heterozygous *Usp9x* ^+*/*−^ females display embryonic lethality [53, 62]. USP9X is required from the earliest stages of development. Depletion of USP9X from two-cell mouse embryos halts development at the blastocyst stage and results in slower blastomere cleavage rate, impaired cell adhesion and a loss of cell polarity [47]. The early requirement of USP9X in embryonic development is also evident in *Drosophila*, where faf is required for the initial cellularisation and polarity of syncytial embryos [15]. At gastrulation, Usp9x is required for mesoderm formation, at least in *Xenopus*, through its regulation of TGFβ signalling. Usp9x promotes TGFβ pathway signalling by deubiquitylating Smad4, allowing it to complex with phosphorylated receptor Smads and then shuttle into the nucleus to execute transcriptional responses to TGFβ family ligands [9]. Faf regulation of TGFβ signalling also regulates threshold responses to morphogens (decapentaplegic) during dorso-ventral patterning in Drosophila embryos [63]. Furthermore, mouse neurons lacking USP9X fail to respond to TGFβ signalling [64]. Intriguingly, USP9X also has the potential to negatively influence TGFβ signalling, via the deubiquitylation and stabilisation of the SMURF1 E3 ligase, which downregulates TGFβ receptors [65].

**Fig. 2:**
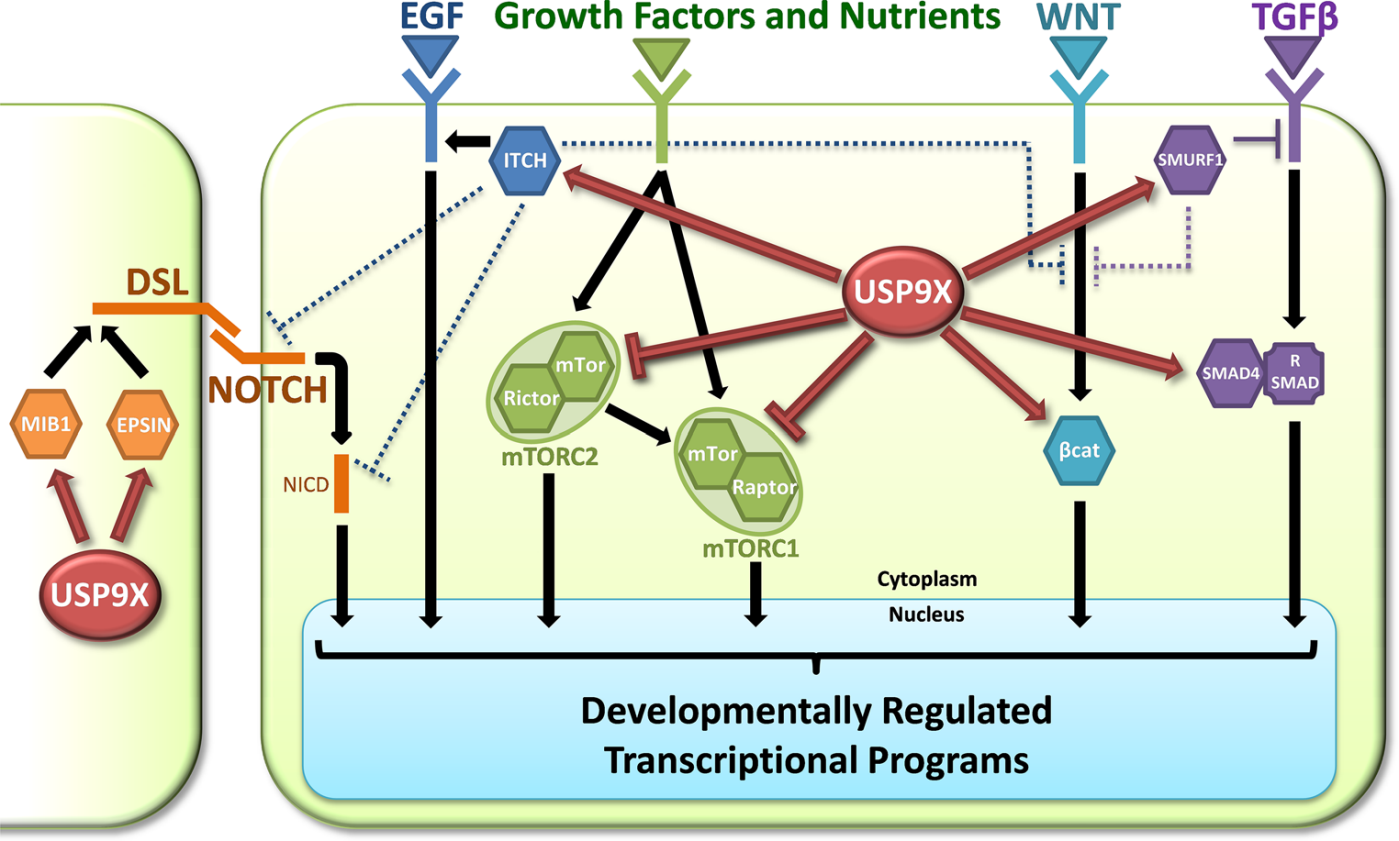
Coordination of developmental signalling pathways by USP9X. Interactors of USP9X (*hexagons*) are key regulators, or signal transduction molecules, of signalling pathways important for many aspects of embryogenesis. In Notch signalling (*orange*), USP9X is required in the signal sending cells to antagonise the proteasomal degradation of both Mind bomb1 (MIB1) and Epsin1, which control key endocytic events of Notch ligands (Delta, Serrate and Lag2; DSL) required for signalling competence. In EGF signalling (*dark blue*), USP9X protects ITCH from proteasomal degradation, which then inhibits the delivery of the EGF receptor to the lysosome and hence promotes signalling output. USP9X interacts with multiple components of the mTOR signalling pathway (*green*) including mTOR, Raptor and Rictor. Although the direct consequence of these interactions is unknown, Usp9x negatively regulates the signalling output of the two mTOR signalling complexes, mTORC1 and 2. USP9X can promote canonical Wnt signalling (*light blue*) by protecting the central signal transduction component β-catenin (βcat) from proteasomal degradation, thus promoting its accumulation, which is associated with nuclear translocation. USP9X can also affect TGFβ signalling (*purple*) both positively and negatively. USP9X protects SMURF1 from proteasomal degradation, which enables it to directly downregulate TGFβ receptors from the cell surface. In contrast, USP9X promotes TGFβ signalling by reversing the effects of mono-ubiquitylation of the common Smad4, which inhibits its ability to form signal transducing complexes with receptor SMADs (R SMAD). Adding further complexity, USP9X substrates can themselves regulate multiple signalling pathways, for example ITCH also negatively regulates Notch and Wnt signalling (*dark blue dotted lines*), whilst SMURF1 also negatively regulates Wnt signalling (*purple dotted lines*)

The regulation of TGF signalling is the best example of how USP9X’s involvement in signal transduction impacts developmental processes. As mentioned earlier, USP9X is also implicated in the Notch, EGF, Wnt and mTOR signalling pathways and therefore is likely to impact many other developmental processes. Its position in these pathways suggests potential coordination of cellular responses to multiple signalling inputs (Fig. 2).

In post-gastrulation murine embryos, ubiquitous Usp9x transcript expression is observed but becomes more complex from mid-gestation until birth. What drives the complex nature of its expression is however largely unknown. USP9X gene expression is reportedly regulated by p53 and p63 [66, 67] and microRNA regulation of USP9X has also been reported [68]. However, it is unclear under what circumstances this regulation occurs and its relevance to developmental expression patterns. In general, high expression of USP9X is maintained in undifferentiated progenitor and stem cells and decreases as differentiation proceeds. For example, USP9X is highly expressed in the nascent limb buds, but its expression is switched off in a proximal to distal pattern as differentiation progresses along the same axis as the limb grows [17]. However, USP9X is also expressed in cells undergoing apoptosis between the digits, indicating that USP9X is not only a stem cell factor. The recent generation of inducible LoxP based *USP9X* knockout mice will be a valuable tool for the ongoing dissection of the role of *USP9X* during development [62, 64].

### USP9X and stem cells

Despite wide variations in cell morphologies and functions, all stem cells are defined by two unique characteristics, their ability to self-renew and differentiate. These signature features have been interrogated using transcriptomic approaches, with the aim of identifying ‘stemness’ genes based on enriched expression in the stem cell population compared to immediately derived differentiated progeny. USP9X has been identified in studies of mouse [69, 70] and human [71] stem cells including embryonic, neural and hematopoietic stem cells and adult epidermal stem cell populations. USP9X was likewise identified using proteomics approaches in both human and mouse ESCs [72, 73]. Consistently, USP9X protein is expressed highly in stem cells in vivo including blastomeres of pre-implantation embryos [47] and neural stem cells [21, 64]. Only recently have studies begun to address the function of USP9X in stem cell populations. Interestingly, deletion of USP9X from mouse ESC did not significantly affect their capacity to be cultured in vitro [53]; however, it may still regulate early differentiation. USP9X has been detected in both Oct4 and Sox2 interaction networks [74, 75]. Regulation of these proteins and networks at the post-translational level by ubiquitylation and deubiquitylation is important for lineage specification. In muscle stem cells, USP9X regulation of the mTOR pathway has been shown to control proliferation and differentiation [76], whilst USP9X levels also control the behaviour of neural stem cells (see below). Thus, the proposal based on expression that USP9X is important for stem cell function is beginning to be realised in multiple types and warrants broader interrogation.

### USP9X in neural development

USP9X is very highly expressed in neural stem cells/progenitors (NSC/NP) in vivo in both mice [17, 21] and zebrafish [14] embryos. In the adult brain, USP9X expression is significantly reduced, but is maintained in the neural progenitors residing in the adult neurogenic niches [21, 64]. USP9X expression levels influence NP organisation and fate both in vivo and in vitro. Nestin-Cre mediated deletion of *Usp9x* from neural progenitors, during mouse central nervous system development, results in a profound disruption of cortical architecture, particularly affecting the proliferative zones containing the NPs [64]. A dramatic decrease in hippocampal size was also prominent, suggesting that hippocampal NPs are particularly sensitive to USP9X function [64]. On the other hand, overexpression of USP9X in cultured, adherent NPs promoted their organisation into polarised clusters, which was associated with increases in their self-renewal capacity [21]. Therefore, consistent with its identification as a stemness gene, USP9X regulates NPs.

USP9X also regulates the growth and function of post-mitotic neurons. Deletion of USP9X results in reduced neuronal outgrowth both in vivo and in cultured hippocampal neurons [18, 64]. In the latter instance, deregulation of TGFβ signalling and cytoskeletal proteins was suggested to underlie the neuronal growth defects (see below). Neuronal overexpression of *faf* induces severe disruption of synaptic growth control at the neuromuscular junctions, evident by an increase in the number of synaptic boutons, elaborate synaptic branching and decreased synaptic transmission. That loss of function *lqf* mutants negate the effects of Faf overexpression on synaptic bouton number suggest deregulated endocytosis may underlie the altered synaptic growth [77]. In other studies, Faf overexpression was found to enhance loss of synaptic boutons resulting from Par1 overexpression, whereas Faf depletion rescued the defect. Further interrogation of this genetic interaction revealed that Faf deubiquitylated phosphorylated Par1, thereby enhancing its activity [78]. These observations indicate that USP9X is involved in multiple aspects of neural development. Not surprisingly, USP9X has now been implicated both directly by mutation or indirectly in a number of human neurodevelopmental and neurodegenerative disorders (see below).

## USP9X in disease

**Fig. 3:**
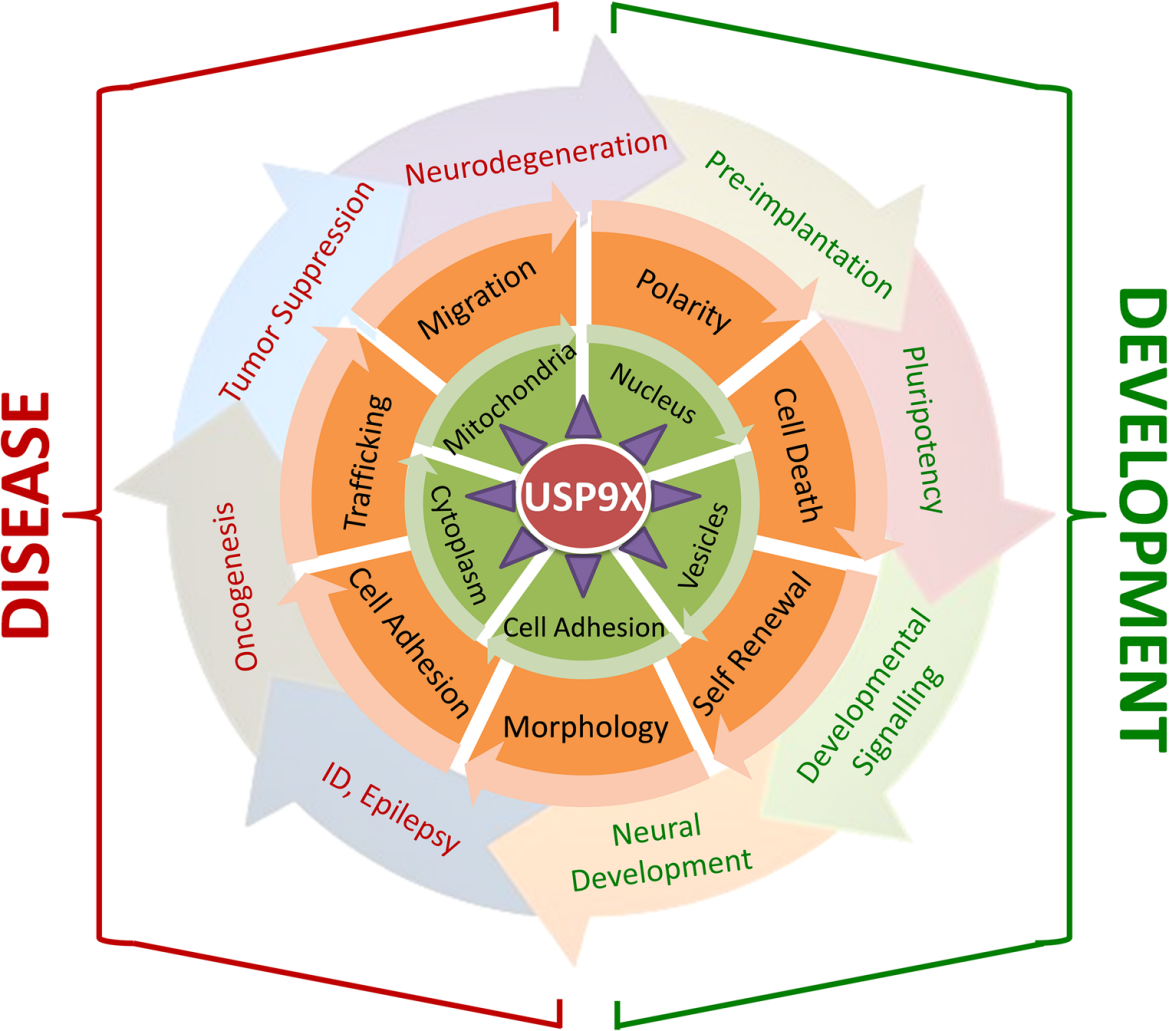
Overview of the contribution of USP9X to development and disease. USP9X (*red*) interacts with 35 known proteins (*purple triangles*), many of which are substrates. The interaction between USP9X and its binding partners is regulated by the sub-cellular localisations of both (*green circle*). The outcomes of USP9X interactions are known to control many cellular processes and behaviour (*orange circle*), which likely underlie the involvement of USP9X in development and disease processes. Each layer of USP9X involvement (i.e. the concentric circles representing substrates, localisation, cell behaviour and developmental and disease processes) can be rotated (*arrowheads*) so as to find combinations of relevant mechanisms. For example, the localisation of USP9X and AF6 at sites of cell–cell adhesion is required for the polarity of blastomeres and pre-implantation development, whilst the interaction of USP9X and MCL1 in the cytoplasm results in the activation of the anti-apoptotic pathways that can lead to tumour resistance against oncogenic therapies

Many of the above descriptions on the molecular, cell and developmental processes regulated by USP9X have also been described in the context of pathological mechanisms underlying various human diseases. In line with these roles, recent investigations of neurodevelopmental and neurodegenerative disorders as well as cancer have revealed the involvement of USP9X (Fig. 3). Investigations of these disorders help in understanding the normal roles of USP9X, and reciprocally appreciating the diverse roles of USP9X will provide insights into disease development.

### USP9X in neurodevelopmental disorders and neurodegeneration

Consistent with its functions during mouse embryonic brain development, USP9X has been implicated in a number of human neurodevelopmental disorders. Three *USP9X* mutations (2 missense and 1 truncating) have been associated with X-linked intellectual disability [18, 79]. Functional interrogation of these mutations revealed that they were loss of function, in that the reductions in axonal growth and neuronal migration observed in mouse neurons lacking *Usp9x* could be rescued by human wild-type *USP9X*, but not the mutated forms [18]. That all three mutations are located in the protein’s C-terminal region suggests it harbours functions important to neural development, and in particular axonal growth and neural migration. Additional indirect evidence for the involvement of USP9X in neurodevelopmental disorders comes from its interaction with doublecortin (DCX). Mutations in *DCX* cause defective neuronal growth and migration leading to lissencephaly in males and sub-cortical band heterotopia in females, with associated epilepsy and intellectual disability features [80, 81]. Importantly, one such mutation in *DCX* specifically disrupts the DCX–USP9X interaction whilst maintaining others, suggesting that the interaction is critical [82]. Reciprocally, the disruption of DCX–USP9X interaction may underlie pathology resulting from *USP9X* mutations; the mutations cluster within the USP9X C-terminus, which is known to bind DCX and mutant USP9X proteins that fail to co-localise with DCX in the axonal growth cones. Intriguingly, both USP9X and DCX knockout mice models display similar neuronal migration defects [64, 83, 84]. Still, other USP9X interactions may also be affected by the mutations. For example, the C-terminus of USP9X is also known to bind to Smurf1 [65], and thus deregulation of TGFβ signalling could similarly contribute to the axonal growth defects, as has been reported in neurons isolated from *Usp9x* knockout mice [64]. With the advance in high throughput genetic sequencing technologies, enquiries into patients with various neurodevelopmental disorders may provide further evidence of the involvement of USP9X [85].

Several lines of evidence suggest that USP9X also plays a significant role in the aetiology of neurodegenerative diseases. The presence of neuronal cytoplasmic inclusions composed of an accumulation of ubiquitylated proteins is a distinctive characteristic of neurodegenerative disorders such as Alzheimer’s, Parkinson’s and Huntington’s disease. Understanding the balance of ubiquitylation and deubiquitylation is critical for treatment. α-synuclein aggregation within Lewy bodies is a pathological hallmark of both Parkinson’s disease (PD) and diffuse Lewy body disorder. In the brain tissues of PD patients, USP9X colocalises with α-synuclein inclusions, and in vitro studies show a functional interaction; whilst monoubiquitylated α-synuclein is degraded by the proteasome, USP9X deubiquitylation of α-synuclein directs its degradation by the less efficient autophagy pathway [86]. In PD patients where proteasomal inhibition is a feature, reduced levels of USP9X is also found, which may explain the accumulation of toxic, monoubiquitylated α-synuclein [87]. In the MPTP (1-methyl-4-phenyl-1,2,3,6 tetrahydropyridine)-induced acute PD mouse model, however, USP9X expression was significantly upregulated in all areas of the brain, perhaps reflecting a cellular response to toxicity [87]. A role for USP9X in the degradation of accumulated proteins has also been suggested in Huntington’s and Alzheimer’s disease. USP9X interacts with Htt from mouse brains of both wild type and YAC128 Alzheimer’s model (transgenic mice expressing human Huntingtin protein) [88]. In a *Drosophila* Alzheimer’s model, overexpression of *faf* was found to enhance toxicity of Aβ-42 expression [78]. Thus, it will be interesting to investigate further the role of USP9X in neurodegenerative diseases characterised by protein accumulation. USP9X has also been linked to spinal muscle atrophy (SMA), a hereditary disorder resulting from reduced expression of survival motor neuron (SMN). Here, USP9X activity is thought to be protective as it directly interacts with SMN in 293T cells protecting it from proteasomal degradation and also regulates the protein levels of core members of the SMN complex [42]. In aggregate, it is clear that USP9X could contribute to the expressivity and outcomes of neurodegenerative disorders; however, the evidence thus far has been mostly associative. Functional interrogation using animal models with supportive human evidence is required to establish the actual role that USP9X may play in neurodegeneration.

### USP9X and cancer

Deubiquitylating enzymes play a major role in homoeostasis and, not surprisingly, are increasingly implicated in the initiation and/or progression of cancer. A comprehensive in situ hybridisation analysis of nine human cancers revealed that several DUBs, including USP9X, are frequently dysregulated in cancers [89]. These results are supported by our queries on the Oncomine Research edition (Table 2) and COSMIC database [90, 91] as well as studies on prostate cancers [92]. USP9X expression is significantly upregulated in ERG-positive prostate tumours compared to ERG-negative and benign tumours [92]. Intriguingly, USP9X deubiquitylates and stabilises ERG protein levels in prostate cancer cells. In human osteosarcoma cell line SaOS2 expressing prostate-specific antigen, USP9X was also significantly upregulated [93]. Together, these (and other) studies reveal that USP9X expression is deregulated in cancers. Such knowledge may be of prognostic value, as an increase in USP9X expression in multiple myeloma patients was associated with poor prognostic outcomes [24], and increased USP9X expression in oesophageal squamous cell carcinoma was correlated with poor survival after radical surgery [94]. Conversely, in pancreatic ductal adenocarcinoma, decrease in USP9X mRNA correlated with poor prognostic outcomes [95]. Recently, it was shown that loss of USP9X function prevented tamoxifen-induced proliferation arrest in oestrogen receptor α-positive breast cancer cells [96].

**Table 2:** Oncomine analysis for USP9X

Type of cancer	Overexpressed	Underexpressed
Cervical cancer	2/5 (40 %)	
Colorectal cancer	6/25 (24 %)	
Lymphoma		8/35 (23 %)
Other cancer	5/29 (17 %)	1/29 (3 %)
Kidney cancer	3/20 (15 %)	
Breast cancer	5/43 (12 %)	3/43 (7 %)
Prostate cancer	2/18 (11 %)	1/18 (6 %)
Brain and CNS cancer	2/26 (8 %)	6/26 (23 %)
Sarcoma	1/17 (6 %)	
Bladder cancer		2/11 (18 %)
Melanoma		1/6 (17 %)
Ovarian cancer		2/13 (15 %)
Lung cancer		3/22 (14 %)
Leukaemia		2/25 (8 %)
Head and neck cancer		1/26 (4 %)

USP9X expression in different cancers (analysis: cancer versus normal). Set threshold *P* value <0.05; fold change >1.5; gene rank, top 10 %. Table represents ratio and percentage for overexpression and underexpression in relation to the total number of analyses available in the database

Consistently, USP9X has been implicated as both an oncogene and tumour suppressor, depending on the type and stage of cancer (see below). Whilst few studies have looked at the transcriptional regulation of USP9X gene expression, it is interesting in this context that *USP9X* is a p53 target [67]. Adding further complexity, a cancerous p53 mutant has been shown to bind USP9X, revealing a potentially interesting regulatory loop [97]. In addition to gene expression changes, exome sequencing and recurrence testing show that *USP9X* is frequently altered in many cancers. Of the 86 cancer types listed in cBioPortal for Cancer Genomics [98, 99], mutations or copy number variations in USP9X were found in 53 types (62 %), with the frequency of *USP9X* within a single cancer type alterations found up to 13 %. *USP9X* alterations are even more frequent in other cancers; for example, it was found in 22 % of gingivo-buccal oral squamous cell carcinoma (OSCC-GB) samples [100]. Comparisons of the frequency of natural variation in *USP9X* alleles derived from control individuals in the EXAC database and cancer samples deposited in the COSMIC database (using the most conservative approach where all samples are assumed male to derive *USP9X* allelic frequencies) reveal that cancer samples have 29 times more synonymous variation, but have 578 times more non-synonymous changes (missense, non-sense and insertion/deletions). Many of these changes are likely to be damaging, for example, whilst non-sense variants are never found in control samples, they are found at a frequency of 1 in every 518 cancer samples. Ongoing large-scale cancer sequencing projects are predicted to reveal further genetic evidence of the involvement of USP9X in cancer.

Whilst the above evidence generally implicates roles in cancer, more detailed functional investigations of USP9X’s molecular interactions provide direct evidence and hints on how *USP9X* can display both oncogenic and tumour suppressor activities. One oncogenic function of USP9X derives from its interaction with the anti-apoptopic protein MCL1, which is highly expressed in cancers and associated with resistance to chemotherapy [101–103]. USP9X deubiquitylation of MCL1 inhibits its proteasomal degradation, thus promoting its anti-apoptotic functions. In human follicular lymphomas, colon adenocarcinoma and small cell lung carcinomas, increased USP9X expression correlates with elevated MCL1 protein levels [24, 104]. Furthermore, the chemical inhibition of USP9X increased the sensitivity of the human lung carcinoma lines A549 and H1299 to an anti-apoptotic inhibitor (ABT-737, targets BCL-xl, but not MCL1). The importance of the MCL1–USP9X interaction extends to other cancer cells. In Jurkat T lymphoma and K562 chronic myelogenous cells, USP9X is enzymatically activated in response to ionising radiation and causes MCL1 stabilisation, in turn inhibiting apoptosis and resulting in radioresistance [105].

Conversely, USP9X has tumour suppressor functions via its genetic interaction with *Kras*. Mutations in *KRAS* are frequently found in pancreatic ductal adenocarcinoma (PDA), and expression of oncogenic *Kras* ^*G12D*^ mutation in mouse pancreatic tissue initiates the development of PDA. In these models, genetic inactivation of *Usp9x* (either by insertional mutagenesis or Pdx1-Cre mediated deletion) was found to enhance oncogenic *Kras* ^*G12D*^ in accelerating tumourogenesis and cancer progression [95]. Surprisingly, the Pdx1-Cre/KRas^G12D^/Usp9x^−/Y^ mice also developed aggressive oral papillomas, likely due to additional Cre recombinase activity in suprabasal keratinocytes [106]. In mouse PDA cell lines, *Usp9x* depletion resulted in increased transformation and decreased anoikis [95]. Whilst the molecular link between *Usp9x* and *Kras* remains to be revealed, reduced expression of the USP9X substrate ITCH was observed in PDA cells lacking USP9X, and ITCH overexpression could rescue the transformation and anoikis phenotype, together suggesting ITCH as a likely mediator of USP9X tumour suppressor function [95]. Both loss of USP9X and ITCH were associated with PDA in humans.

Overall, the evidence suggests that the role of USP9X in cancer is tissue specific and implicates USP9X as a clinically relevant candidate warranting further investigation.

## Conclusions and future directions

The regulation of various proteins by USP9X is a complex and dynamic process that is central to cellular processes including cell adhesion, polarity, vesicular trafficking, signal transduction and apoptosis. Through these roles, USP9X has been found to regulate developmental processes, and deregulated USP9X function is implicated in neurodevelopmental and neurodegenerative diseases as well as various cancers. Whether such knowledge can translate into clinical practice remains unanswered. Addressing this question is challenging and will require reciprocal investigations between the normal and pathological roles of USP9X.

One major challenge will be to understand the developmental, cellular (including subcellular) and molecular context of USP9X-regulated processes. USP9X interacts with a large number of substrates and has remarkable potential to coordinate the inputs of multiple signalling pathways with cellular responses. However, its requirement in any process will be underpinned by the context in which it exists. This fact is highlighted by the apparent contradiction is some reported USP9X roles, for example, both promoting and inhibiting TGFβ signalling, acting in both pro- and anti-apoptotic pathways and displaying both oncogenic and tumour suppressor functions. Dissecting which particular interactions (or sets of interactions) are critically required for any given processes and in what context will be crucial. This will require an in-depth understanding of what governs the ability of USP9X to interact with substrates and proteins, and involve resolution of its protein structure. The complex and dynamic expression of USP9X and its substrates suggests that this task will be a daunting one, but investigations of the effects of rare genetic variants associated with neurological disorders have begun to provide important insights and should remain a focus of future research.

The second major challenge will be the manipulation of USP9X in a context-specific manner to provide therapeutic avenues. Current approaches have employed WP1130, a small molecule DUB inhibitor that targets USP9X along with USP5, USP14 and UCH-L5 [107]. However, the mechanism of WP1130 inhibition of DUBs is currently unknown and there are no USP9X-specific inhibitors. Furthermore, such an approach lacks the required contextual specificity and, whilst perhaps offering beneficial disruption of a disease-relevant process, will likely be detrimental to other USP9X-regulated processes leading to unwanted side effects. Possible solutions to this problem will lie in the discovery of what regulates USP9X expression, sub-cellular localisation and enzymatic activity in particular cells. For example, it is known that USP9X utilises alternative 3′UTR sequences in response to neuronal activity [108], and this might provide a specific miRNA-based therapeutic target for treatment in neurological disorders such as epilepsy. Likewise, the transcription factor p53 has been reported to influence USP9X expression [67], which might provide an avenue to target certain cancers. Post-translational modification of USP9X such as phosphorylation might also offer new approaches to alter its activity in specific contexts. Once again, these knowledge gaps should be approached by studying the involvement of USP9X in normal and diseased states.

The extraordinary level of conservation and involvement in fundamental cell and developmental processes have identified USP9X as a molecule of great importance. The complexities of its function and regulation will be challenging to dissect; however, this accomplishment will be accompanied by new exciting insights into human biology and disease.
